# Production loss and sick leave caused by antibiotic resistance: a register-based cohort study

**DOI:** 10.1186/s12889-022-12947-x

**Published:** 2022-03-17

**Authors:** Sofie Larsson, Mikael Svensson, Anders Ternhag

**Affiliations:** 1 Public Health Agency of Sweden, SE–171 82 Solna, Sweden; 2 School of Public Health and Community Medicine, Institute of Medicine, University of Gothenburg, Gothenburg, Sweden; 3 Department of Medicine, Solna, Karolinska Institute, Stockholm, Sweden

**Keywords:** Societal costs, Production loss, Antibiotic resistance, Days of sick leave, Two-part model

## Abstract

**Background:**

Adverse economic consequences of antibiotic resistance, both in health care systems and in society at large, have been estimated to emerge and significantly affect the global economy. To date, most studies of the societal costs of antibiotic resistance have had a macroeconomic perspective, using the number of attributable deaths as a quantifier for production loss. In contrast, there have been few studies of the consequences of antibiotic resistance in terms of the length of sick leave and hence the impact of morbidity on production loss. The aim of our study was to estimate the production loss from ill health caused by antibiotic resistance.

**Method:**

To estimate additional production loss due to antibiotic resistance, we used Swedish register-based cohort data to determine days of long-term sick leave (LTSL) for episodes of infection caused by resistant and susceptible bacteria respectively. We collected patient data for four common infection types (bloodstream infection, urinary tract infection, skin and soft tissue infection, and pneumonia), as well as, antibiotic susceptibility test data, and total days of LTSL. We used a two-part model to estimate the number of LTSL days attributable to resistance, and controlled for comorbidities and demographic variables such as age and gender.

**Results:**

The results show that antibiotic resistance adds an additional 8.19 days of LTSL compared with a similar infection caused by susceptible bacteria, independent of infection type and resistance type. Furthermore, the results suggest that production loss due to temporary sick leave caused by antibiotic resistance in a working-age population amounts to about 7% of total health care costs attributable to antibiotic resistance in Sweden.

**Conclusion:**

Estimating the effect of antibiotic resistance in terms of temporary production loss is important to gain a better understanding of the economic consequences of antibiotic resistance in society and, by extension, enable more effective resource allocation to combat further emergence of resistance. Society’s economic costs of antibiotic resistance are, however, probably much greater than those of sick leave due to disease alone.

**Supplementary Information:**

The online version contains supplementary material available at 10.1186/s12889-022-12947-x.

## Background

Antibiotic resistance (ABR) has increased globally over several decades, resulting in a constant need for new antibiotic drugs that are effective against resistant bacteria [1]. However, the development of new antibiotics faces major challenges of both scientific difficulties and high development costs that make other interventions to curb resistance even more important. Resources are already invested in various antibiotic stewardship programs aimed at enhancing the use of antibiotics in, for example, diagnostics, choice of drug, and duration of treatment to reduce or slow down development of resistance [2]. To guide the allocation of resources to combating ABR, there is also a need for accurate estimates of the total burden of resistance, including estimates of the economic impact both on the health care sector and on society.

In 2014, the World Health Organization (WHO) conducted a systematic review that included studies on economic burden and cost differences related to ABR [3]. The review showed that studies quantifying the consequences of ABR were limited. These had concluded, for example, that impact on society had not been adequately measured, and highlighted this as an important knowledge gap for future research to fill. Another report, on the public health and economic consequences of antimicrobial resistance (AMR), from the Organization for Economic Co-operation and Development (OECD) in 2018 [4], concludes that the lack of a broader societal perspective is a common limitation of economic evaluations of AMR.

Results from studies that included societal costs in their economic evaluations of AMR indicate that these costs may amount to more than 40% of the total costs of resistance [5–10]. However, methods of estimating societal costs differ significantly among the studies. A study by Roberts et al. [10], for example, based estimates of societal costs on production loss due to the numbers of deaths attributable to AMR, compared with other illnesses. Since AMR was compared with other illnesses it could not simply be assumed that the total costs were attributable to resistance alone; rather, they were thought to be due to *infections* caused by antimicrobial resistance. This was criticized by the WHO in its systematic review [3].

In one of the most cited reports in recent years, the O’Neill review from 2016 [8], the global economic consequences of deaths attributable to AMR was estimated to be about 100 trillion dollars up to 2050, which would significantly reduce the world’s Gross Domestic Product (GDP) [7–9, 11]. This analysis was based on projected number of deaths among people of working age [11]. However, since the impact was estimated at a global macroeconomic level (i.e. in terms of global GDP), the results cannot be used to estimate individual effects on societies if the risk of dying from infection varies at national and/or regional level.

Economic evaluations of interventions are often conducted as cost-effectiveness analyses (CEA). Depending on the perspective of the study, i.e. whether the focus is on health care perspective or societal perspective, these analyses include different types of cost. Both perspectives include health care costs, whilst the societal perspective also includes other costs unrelated to health care, such as cost of production loss or costs to other sectors [12, 13]. Although several studies have calculated the health care costs of ABR, studies on the societal costs of ABR remain scarce and most analyses are based solely on assumptions of potential production loss due to resistance. Societal costs are often described as the resources lost, indirectly, due to illness or treatment, such as absence from work or decreased consumption. The aspect most frequently referred to in CEAs is production loss, i.e. time absent from work [14]. Production loss could therefore be due to both mortality and morbidity.

Overall, estimates of the societal costs of ABR are lacking, and most of the studies published to date are based solely on assumptions about the magnitude of production loss due to infection caused by ABR. The aims of this study were to quantify the additional days’ production loss attributable to ABR, by using register data on individuals’ sick days at the time of infection, and to compare cases of infection caused by antibiotic-resistant bacteria with those caused by susceptible bacteria.

## Methods

### Data

To identify the study population, we used the 10th edition of the *International Classification of Diseases* (ICD-10) codes of relevant infections recorded in the patient register (Table 1) held by the Swedish National Board of Health and Welfare (NBHW) [15]. The patient register contains data on every in- and out-patient health care visit in Sweden, together with data on patient age, sex, and comorbidities. Data concerning all patients up to the age of 65 were collected from 2011 to 2015. The ICD-10 codes [16] were categorized by type of clinical infection (bloodstream infection, BSI; urinary tract infection, UTI; skin and soft tissue infection, SSI; and pneumonia); see Table 1. Data on hospital admissions, visits to specialists, and all diagnoses registered at the time of the patients’ visits and/or tests were collected from the patient register [15].

**Table 1:** ICD-10 codes grouped by clinical infection type

Infection type	Bloodstream infection	Pneumonia	Urinary tract infection	Skin and soft tissue infection
ICD-10-SE code [16]	A40, A41, I30, I33, K35, K57, K81, P36, T80.2, T81.4, T82.2, T82.7, T84.5, T84.6, T85.7	H66.0, H66.4, H66.9, J01, J13, J15, J18.9	N10, N11, N12, N30, O86.2	A46, L02, L03, L08, L97, L98.4

To identify cases with ABR, we compared infection cases from the patient register with cases reported in the communicable disease database (SmiNet) held by the Public Health Agency of Sweden (PHAS) [17]. PHAS has national responsibility for surveillance and prevention of communicable diseases and other public health threats. We used data for the five types of resistance that are notifiable under the Communicable Disease Act in Sweden [18]: methicillin resistant *Staphylococcus aureus* (MRSA); extended-spectrum beta-lactamase (ESBL) in Enterobacterales*;* carbapenemase-producing Enterobacterales (CPE); penicillin-non-susceptible *Pneumococci* (PNSP); and vancomycin-resistant *Enterococci* (VRE). All detected cases of these types of resistance are reported by laboratories and clinicians, along with data on patient age, gender, and specimen type, in the SmiNet database [17]. The use of personal identity numbers in Sweden has enabled entries in different registers to be merged and the registers linked at individual level.

In Sweden, people of working age are insured through the social insurance system, independent of employment status. Accordingly, anyone who falls ill is assured of sickness benefit. If the person is employed, the employer is liable to pay the sickness benefit for the first 14 days. For the unemployed, sickness benefit is paid by the Swedish Social Insurance Agency (SSIA) instead. Sickness benefit in Sweden is approximately 80% of the individual’s daily salary, subject to a cap of close to EUR 80 or EUR 54 daily, depending on employment status [19]. After 14 days’ sick leave, the SSIA pays this benefit irrespective of employment status. This phase is referred to as “long-term sick leave” (LTSL). Only sickness benefit paid by the SSIA is registered in the national sick-leave register [20], which means that information on sickness benefit paid by the employer is lacking if the employee’s total absence from work lasts less than 14 days.

**Fig. 1:**
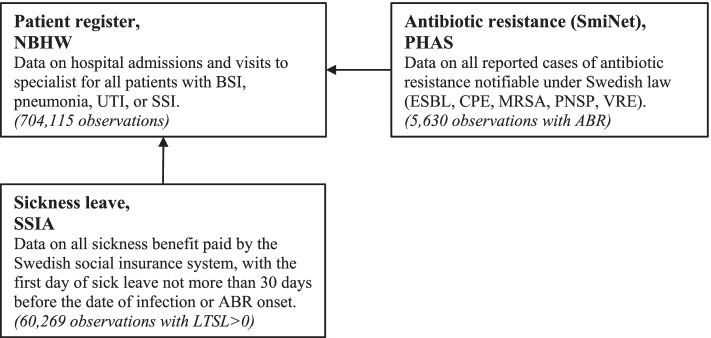
Flow-chart of how register data was matched

For every observation of ABR or infection, we matched data on the number of LTSL days from the SSIA if the date of testing or other contact with the health care system due to infection was within the period of sick leave, or if the leave started within 5 days of the infection onset. Since this matching method potentially includes sick leave lasting several months, we omitted all observations where the first day of sick leave occurred more than 30 days before the infection or resistance was registered. We chose this 30-day limit to include patients receiving hospital treatment who potential were infected during their stay. Observations of ABR or infection not included in the SSIA register were assumed to have zero days of LTSL, since this register contains all LTSL data. Figure 1 is a flow chart showing how matching between the registers was done.

To estimate comorbidity, we calculated the Charlson Index [21, 22] for each individual, using all registered ICD-10 codes in the patient register from 2011 to 2015.

The study was approved by the Ethical Review Board in Stockholm (ref. 2016/2166–31). Data used is not publicly available.

### Statistics

Descriptive statistics, such as mean values and quartile ranges, were calculated for the population based on the cases (with ABR) and controls (without ABR). We estimated production loss as the number of LTSL days due to ABR, independent of resistance type, since samples of CPE, PNSP, and VRE were too small to divide into specific resistance types. In the regression analysis, we used net days of LTSL, taking part-time sick leave into account, and controlled for age, gender, year of infection, comorbidities, infection type, and number of simultaneous infections. Since certain comorbidities are associated both with risk of having an ABR infection (exposure) and with length of sick leave (outcome), i.e. confounders, we included comorbidity as a covariate.

Since the dependent variable was skewed to the right, with many observations of zero days’ sick leave, we conducted a two-part model (TPM) analysis, which is a common method if data is skewed and with many zeros [23]. The first part of the analysis involved estimating the probability that sick leave days were more than zero, using a logistic regression. In the second part, we used a Negative Binomial regression to estimate the proportional change in the dependent variable from a one-unit change in the independent variables, i.e. the Incident Risk Ratios (IRR). IRR was calculated by taking the exponential of the coefficient. Negative binomial regression is often used for over-dispersed count data [24], as we have in our case. Results from the first and second parts were combined in estimates of the marginal effects of each independent variable, by capturing the combined result from the first and second parts of the model.

In both parts, we used robust standard errors clustered by patient ID, since patients might recur in data sets several times if they had been admitted to hospital more than once during the five-year period of data collection.

We tested several model specifications, gradually adding more variables. Except a dummy variable of ABR, our preferred model specification included, at least, [1] the Charlson Index, since we believe that comorbidity may affect both the number of LTSL days and the risk of getting an infection caused by ABR, and [2] a binary variable that takes the value of 1 if the patient had more than one infection at a time. All models included age, gender, and year of infection. We also used the Akaike Information Criterion (AIC) and Schwarz’s Bayesian Information Criterion (BIC) to test the relative goodness-of-fit of the model specifications [25].

In a sensitivity analysis, we ran the above analyses, using total sick leave days instead of net days, to control for differences in how days of sick leave are reported in the register. However, since data on total days do not take part-time sick leave into account, and this analysis potentially overestimated subjects’ actual time away from work, we used only information from these regressions to test the robustness of our results. As a further robustness check, we also ran the TPM with Poisson regression on net days of LTSL, with the same model specification as in the second part of the two-part model.

## Results

### Study participants

From the patient register, we extracted 704,115 hospitalization episodes of infection in patients aged 16–65 years with at least one of the following infection types: bloodstream infection (BSI), urinary tract infection (UTI), skin and soft tissue infection (SSI), and pneumonia. Matching with data from SmiNet, we found that 5630 of total observations, i.e. less than 1% of the sample, also had at least one resistant bacterium. These were considered ‘cases’, while individuals not included in the SmiNet database were considered ‘controls’ (698,485 observations). From the register on sickness benefit, we matched days of LTSL to 60,269 observations, of which 665 were resistance cases. In total, 11.8% of cases had at least 1 day of LTSL, while the corresponding percentage was 8.5% for controls. Observations without information on sick leave were set to zero days since the SSIA register contains all LTSL nationally.

### Descriptive data and outcomes

Table 2 presents descriptive statistics of patients by resistance status. The groups’ average ages were similar: 43.0 years for patients with an infection caused by ABR (cases) and 44.9 years for patients without resistance (controls), 61.3% and 53.9% of the cases and controls respectively being females. The distribution of infection types differed between cases and controls (BSI: 17.2% vs. 38.5%; UTI 60.9% vs. 13.1%; SSI: 22.9% vs. 24.8%; pneumonia: 6.7% vs. 25.4%). Charlson Index was, in general, higher in patients with infection caused by ABR. For cases, the most common resistance type was ESBL followed by MRSA.

**Table 2:** Descriptive statistics for episodes of infection in 2011–2015, by resistance status

	Resistance	No resistance
Age, mean (q1–q3)	43.0 (29–57)	44.9 (33–58)
Female sex, %	61.3	53.9
Charlson Index score, mean and distribution (%)	0.969	0.828
0	67.0	69.2
1	10.8	11.8
2–3	13.7	12.1
4–5	3.2	3.0
≥6	5.3	3.9
No. of days of LTSL, mean (q1–q3)	15.9 (0–0)	5.72 (0–0)
Type of infection, %		
Bloodstream infection	17.2	38.5
Urinary tract infection	60.9	13.1
Skin and soft tissue infection	22.9	24.8
Pneumonia	6.7	25.4
Resistance types, %		
ESBL	76.8	
CPE	0.4	
MRSA	21.8	
PNSP	1.4	
VRE	0.6	

q1: quartile 1, q3: quartile 3

The number of net days of LTSL averaged 15.9 days for the case group and 5.72 days for the control group. For both groups, however, the median number of days was zero, ranging from 0 to 1683 days for cases and 0 to 2277 days for controls. This indicates that days of LTSL were highly skewed to the right, with many zero observations for both groups.

### Regression analysis

In Table 3, we present results from both the first and second parts of the two-part model. We studied four different model specifications, adding more variables for each specification. Model 4, the fully extended model, was our preferred specification since it took into account both comorbidities and the numbers and types of infections. This was also the model with the best statistical fit, in terms of AIC and BIC results (see Table A1 in online appendix). Below, in our interpretations of the results, we therefore focus on the results from model 4.

**Table 3:** Results from the two-part regression model

	TPM Model 1	TPM Model 2	TPM Model 3	TPM Model 4
**Part 1: Odds ratio (OR)**				
Antibiotic resistance	1.46^***^ (0.06)	1.51^***^ (0.06)	1.43^***^ (0.06)	2.23^***^ (0.10)
Age	1.01^***^ (0.00)	1.01^***^ (0.00)	1.01^***^ (0.00)	1.01^***^ (0.00)
Female sex	1.04^**^ (0.01)	1.02 (0.01)	1.02 (0.01)	1.09^***^ (0.01)
Year	0.97^***^ (0.00)	0.97^***^ (0.00)	0.97^***^ (0.00)	0.97^***^ (0.00)
Charlson index		0.86^***^ (0.00)	0.86^***^ (0.00)	0.87^***^ (0.00)
At least two infections at once			2.01^***^ (0.06)	0.93^*^ (0.03)
Bloodstream infection				2.74^***^ (0.06)
Pneumonia				2.75^***^ (0.06)
Skin and soft tissue infection				1.65^***^ (0.04)
Urinary tract infection (reference case)				–
Constant	2.18e+ 25^***^ (1.82e+ 26)	1.10e+ 28^***^ (9.09e+ 28)	3.82e+ 28^***^ (3.17e+ 29)	2.36e+ 24^***^ (1.96e+ 25)
**Part 2: Incidence Rate Ratios (IRR)**				
Antibiotic resistance	2.01^***^ (0.14)	1.88^***^ (0.14)	1.85^***^ (0.14)	1.84^***^ (0.15)
Age	1.02^***^ (0.00)	1.01^***^ (0.00)	1.01^***^ (0.00)	1.01^***^ (0.00)
Female sex	0.88^***^ (0.02)	0.89^***^ (0.02)	0.89^***^ (0.02)	0.91^***^ (0.02)
Year	1.04^***^ (0.01)	1.06^***^ (0.01)	1.06^***^ (0.01)	1.06^***^ (0.01)
Charlson index		1.33^***^ (0.01)	1.33^***^ (0.01)	1.33^***^ (0.01)
At least two infections at once			1.37^***^ (0.07)	1.32^***^ (0.09)
Bloodstream infection				1.09 (0.05)
Pneumonia				0.79^***^ (0.04)
Skin and soft tissue infection				1.06 (0.06)
Urinary tract infection (reference case)				–
Constant	0.00^***^ (0.00)	0.00^***^ (0.00)	0.00^***^ (0.00)	0.00^***^ (0.00)
Observations	704,115	704,115	704,115	704,115

Exponentiated coefficients; Standard errors in parentheses

First part: Logistic regression, Second part: Negative Binomial regression

^*^p < 0.05, ^**^ *p* < 0.01, ^***^ *p* < 0.001

#### Part 1

In the first part of the two-part model, the odds ratio (OR) of having at least 1 day of LTSL, for patients with ABR compared with controls, is the exponential of the coefficient, i.e. exp.(0.80) = 2.23. Results presented as OR in Table 3 suggest that the odds of having at least 1 day of LTSL was 2.23 times higher if the patient had an infection caused by ABR compared to if it was an infection caused by susceptible bacteria, indicating that it was more likely for patients with ABR to have LTSL> 0 days. The odds of having some LTSL decreased by about 13% (OR = 0.87), with an increase in the Charlson Index. Being female increased the odds of having LTSL.

#### Part 2

In the second part, we estimated the effect of a one-unit change in variables, i.e. the Incident Risk Ratios (IRR), on the number of days, given that LTSL was at least 1 day. Results in Table 3 suggest that ABR infection increase days of LTSL by 1.84 times, compared with infections caused by susceptible bacteria. Furthermore, age and year of infection had positive effects on the number of days, while female gender had a negative effect. The number of days increased by about 1.33 times when the Charlson Index increased by one, and with 1.32 times for patients who had more than one infection at once.

### Marginal effects

Marginal effects were calculated for the whole sample, thus giving the average effect of variables included on the number of net days of LTSL. Results presented in Table 4 show that the average effect of ABR on LTSL was approximately 8.19 days per infection period. A higher Charlson Index, indicating more severe comorbidities, led to 0.95 additional days of LTSL on average, as the Charlson Index increased by 1. Having more than one infection resulted in a marginal effect of 1.31 day more of LTSL than having only one infection. The number of LTSL days due to infection type was between 3 and 6 more days’ LTSL compared with UTI, the reference case.

**Table 4:** Marginal effects from two-part regression model

	TPM Model 1	TPM Model 2	TPM Model 3	TPM Model 4
Antibiotic resistance	6.12^***^ (0.48)	6.12^***^ (0.53)	5.73^***^ (0.53)	8.19^***^ (0.56)
Age	0.15^***^ (0.01)	0.14^***^ (0.01)	0.14^***^ (0.01)	0.12^***^ (0.01)
Female sex	−0.56^**^ (0.17)	−0.61^***^ (0.17)	−0.59^***^ (0.17)	−0.09 (0.17)
Year	0.08 (0.06)	0.16^**^ (0.06)	0.16^**^ (0.06)	0.16^**^ (0.06)
Charlson index		0.92^***^ (0.07)	0.90^***^ (0.07)	0.95^***^ (0.07)
At least two infections at once			5.83^***^ (0.35)	1.31^**^ (0.46)
Bloodstream infection				6.16^***^ (0.35)
Pneumonia				4.14^***^ (0.36)
Skin and soft tissue infection				3.16^***^ (0.37)
Urinary tract infection (reference case)				–
Observations	704,115	704,115	704,115	704,115

Standard errors in parentheses

^*^ *p* < 0.05, ^**^ *p* < 0.01, ^***^ *p* < 0.001

### Sensitivity analysis

In a sensitivity analysis, we checked the robustness of the results by using total days of LTSL. The results from this analysis showed the same relative change in the effect of ABR on LTSL as in our main analysis. As a further robustness check, we also ran a TPM with Poisson regression in the second part. This analysis too showed robustness in our results. Sensitivity analyses are presented in an online appendix, Tables A2–A5.

## Discussion

Results from this analysis suggest that ABR causes an additional 8.19 days of LTSL compared with a similar infection caused by susceptible bacteria, independent of infection type. As expected, a higher Charlson Index and simultaneous infections increased the number of LTSL days as well. Results concerning the number of additional LTSL days for patients with an infection caused by ABR were used to calculate total production loss due to morbidity from ABR, adding a partial societal perspective to calculations of the number of ABR cases.

From a Swedish perspective, we estimate the production loss due to morbidity caused by ABR at approximately EUR 1.3 million annually,^1^ based on the number of ABR cases in hospital settings between 2011 and 2015. This estimate amounts to about 6.8% of estimated annual health care costs in Sweden due to ABR, totaling approximately EUR 19 million (average health care costs in 2012–15) [28]. However, this is probably an underestimate of the true production loss due to ABR, since the calculation takes into account only morbidity for those in hospital care setting aged 20–64. To estimate the total production loss due to ABR, production loss due to care of a sick child, or due to mortality should also be included, as well as production loss for the less severe cases in the primary care setting.

The methodology of measuring societal costs and production losses due to ABR differs in previous studies. Roberts et al. [10], for example, calculated production loss on the basis of the number of deaths attributable to infections caused by antibiotic resistance. However, this approach does not take into account the fact that there could be an effect of both ABR and the infection, and that these are indistinguishable. Furthermore, as the authors stress, they did not take into account production loss from morbidity or reduction in quality of life. Altogether, it may be argued that this study overestimates the impact on productivity of ABR-attributable deaths, while underestimating the impact from morbidity. In a joint analysis by the European Centre for Disease Prevention and Control (ECDC) and the European Medicines Agency (EMA) from 2009 [6], societal costs for European countries were estimated using both mortality and morbidity. Production loss due to morbidity was estimated by using extra hospital days due to infections caused by resistant bacteria, compared with infections caused by susceptible bacteria. The effect of mortality on production loss was estimated as the foregone earnings from the number of premature deaths associated with resistance.

Differences in methodology depend on which costs are assumed to be attributable to ABR — whether all costs of infection or just the additional costs compared with other infection not caused by resistance — and also which costs that are included as societal costs. In 2013 Smith and Coast [29] argued that the true economic burden of resistance should also consider the burden to society of not having effective antibiotics, potentially leading to canceled surgeries and a major negative impact on quality of life if ‘simple’ infections are no longer treatable. However, to the authors’ knowledge, cost estimates of these kinds of events are still lacking.

In our analysis, we focused on the additional production loss due to resistance, in comparison to analyses that have used total effects of infections with resistance compared with other diseases. We believe that using additional production loss due to ABR is superior for estimating the effects of ABR, rather than the underlying infection. Some argue, however, that using only the additional costs related to resistance leads to excessively modest estimates of total economic burden, since it is not possible to account for relevant but indirect costs due to resistance, such as cost of postponed surgery and other plausible events, if antibiotics lose their effect when used for prophylactic purposes [29].

In 2019 the SSIA paid sickness benefit for more than 55.7 million net days of LTSL to Swedish residents [30]. Our estimates of the numbers of days’ sickness due to MRSA, ESBL, CPE, PNSP, and VRE imply that less than 0.02%^2^ of annual net days of LTSL, at national level, was due to ABR in our low-resistance setting. Furthermore, our estimates of 8.19 days due to ABR means that, on average, everyone aged 20–64 in Sweden would have 0.002 days^3^ of annual sick leave related to resistance, compared with an average of 9.51 days for all types of illness [30, 31].

There are some limitations to this study. First, we include only LTSL, which means that we have no information on production loss if sick leave was shorter than 14 days. Instead, we assumed that these infection episodes had zero days of LTSL, which probably led to underestimation of the total effect on sick leave of infections caused by resistant or susceptible bacteria. However, since we were interested in the additional days, i.e. the difference between infections caused by resistant or susceptible bacteria, and since we studied the effect in hospital patients only, we believe that the differences are relatively small during these first 14 days.

Second, in this analysis, we focused only on the most severe cases, and these patients were cared for in a hospital setting. Accordingly, we disregarded the effect on LTSL of individuals who were in the primary care setting only. The result is potential overestimation of the average number of sick days if our results are used to estimate the overall effect of resistance in terms of production loss. Furthermore, we focused only on production loss due to potential absence from work in a working-age population, defined as infections in persons aged 16–65. With this perspective, we excluded potential production loss due, for example, to an adult caring for a sick child or to infections in older adults (aged over 65) outside the workforce.

This study was performed in Sweden with its specific terms of sick-leave compensation, which could affect the number of sick-leave days, and hence, the production loss. In order to generalize our results to other countries or settings, one might need to consider if and how accessible sick-leave compensation is in different countries, as this could impact the magnitude of LTSL days. However, there is no reason to believe that sick leave in Sweden is paid for longer than necessary, because of the terms of sick-leave compensation. Our results therefore applies to other countries with similar societal insurance systems.

For individuals who are not of working age, production loss is not usually included in the analysis. However, this has been criticized since retirees often contribute with informal production, which should be valued and included in the health economic analysis as well [16]. When an analysis includes children, it is common to include production loss for parents. A cost-effectiveness analysis including production loss for individuals under 65 only, could mean that interventions aimed at the over-65s may be less cost-effective, and thus be given lower priority. The results of our analysis may serve as an approximation both for children and for retirees if there is no reason to believe that these groups differ substantially in length of sickness from the population included in this study.

## Conclusion

The economic consequences of ABR on society most likely exceed the effects of morbidity alone. Our results suggest that production loss from temporary sick leave caused by ABR, in a working-age population, amounts to about 7% of total ABR-related health care costs in Sweden, compared with estimates of production loss at approximately 40% of total costs, i.e. health care and societal costs combined. However, by estimating the effects on production loss of ABR-related morbidity, this study contributes one piece of the puzzle of estimating total societal costs. For an understanding of the bigger picture, further research is needed on other types of resistance, and also sickness absence in other populations such as children, retirees, or less severely sick individuals.

Recent studies on the effects of ABR on production loss are largely based on assumptions alone. Since our results are based on register data, these may help to reduce uncertainty in estimates generally.

## Supplementary Information

**Additional file 1.** Online Appendix.

## Data Availability

The data that support the findings of this study are available from the Public Health Agency of Sweden, National Board of Health and Welfare, and Swedish Social Insurance Agency, but restrictions apply to the availability of these data, which were used with an ethical approval for the current study, and so are not publicly available. Data are however available from the corresponding author (Sofie Larsson) upon reasonable request and with permission of each holder of the registers.

## Abbreviations

ABR: Antibiotic resistance
AIC: Akaike Information Criterion
AMR: Antimicrobial resistance
BIC: Schwarz’s Bayesian Information Criterion
BSI: Bloodstream infection
CEA: Cost-effectiveness analysis
CPE: Carbapenemase-producing Enterobacterales
ECDC: European Centre for Disease Prevention and Control
EMA: European Medicines Agency
ESBL: Extended-spectrum beta-lactamase (in Enterobacterales)
GDP: Gross domestic product
IRR: Incidence Rate Ratios
LTSL: Long-term sick leave
MRSA: Methicillin resistant *Staphylococcus aureus*
NBHW: National Board of Health and Welfare
OECD: Organization for Economic Co-operation and Development
OR: Odds ratio
PHAS: Public Health Agency of Sweden
PNSP: Penicillin non-susceptible *Pneumococci*
SSI: Skin and soft tissue infection
SSIA: Swedish Social Insurance Agency
TPM: Two-part model
UTI: Urinary tract infection
VRE: Vancomycin-resistant *Enterococci*
WHO: World Health Organization

## Footnotes

1 8.19 LTSL days x number of annual infections × 122.5 EUR/day in cost of production loss (average wage and social security contributions [26, 27]).

2 Total annual number of LTSL days due to ABR divided by total number of LTSL days due to any illness.

3 Total annual number of LTSL days due to ABR divided by total number of Sweden’s residents of working age (20–64 years).
