# Supplementary material for: Production loss and sick leave caused by antibiotic resistance: a register-based cohort study
Source: BMC Public Health. 2022 Mar 17;22:527. doi: 10.1186/s12889-022-12947-x (PMC8932015; doi:10.1186/s12889-022-12947-x)
Supplement: Supplementary file 1 — Additional file 1. Online Appendix. [file 12889_2022_12947_MOESM1_ESM.pdf]

Production loss and sick leave caused by antibacterial resistance – a register-based cohort study

Sofie Larsson, Mikael Svensson, Anders Ternhag

Online Appendix

Tables

Table A1 Akaike Information Criterion (AIC) and Schwarz's Bayesian Information Criterion (BIC) to test the relative goodness-of-fit of TPM model specifications

Table A2 Sensitivity analysis: First and second part of TPM on total days of LTSL

Table A3 Sensitivity analysis: Marginal effects from TPM on total days of LTSL

Table A4 Sensitivity analysis: First and second part of TPM with Poisson regression on net days of LTSL

Table A5 Sensitivity analysis: Marginal effects from TPM with Poisson regression on net days of LTSL

Table A1 Akaike Information Criterion (AIC) and Schwarz's Bayesian Information Criterion (BIC) to test the relative goodness-of-fit of TPM model specifications

TPM model specifications	Obs.	AIC	BIC
Model 1	704,115	1,036,683	1,036,798
Model 2	704,115	1,024,211	1,024,349
Model 3	704,115	1,023,357	1,023,518
Model 4	704,115	1,016,693	1,016,922

Table A2 Sensitivity analysis: First and second part of TPM on total days of LTSL

	SA: TPM Model 5	SA: TPM Model 6	SA: TPM Model 7	SA: TPM Model 8
**Part 1: Odds ratio (OR)**				
Antibiotic resistance	1.46***	1.51***	1.43***	2.23***
	(0.06)	(0.06)	(0.06)	(0.10)
Age	1.01***	1.01***	1.01***	1.01***
	(0.00)	(0.00)	(0.00)	(0.00)
Female sex	1.04**	1.02	1.02	1.09***
	(0.01)	(0.01)	(0.01)	(0.01)
Year	0.97***	0.97***	0.97***	0.97***
	(0.00)	(0.00)	(0.00)	(0.00)
Charlson index		0.86***	0.86***	0.87***
		(0.00)	(0.00)	(0.00)
At least two infections at once			2.01*** (0.06)	0.93* (0.03)
Bloodstream infection				2.74***
				(0.06)
Pneumonia				2.75***
				(0.06)
Skin and soft tissue infection				1.65*** (0.04)
Urinary tract infection (reference case)				–
Constant	2.18e+25***	1.10e+28***	3.82e+28***	2.36e+24***
	(1.82e+26)	(9.09e+28)	(3.17e+29)	(1.96e+25)
**Part 2: Incidence Rate Ratios (IRR)**				
Antibiotic resistance	1.81***	1.70***	1.68***	1.66***
	(0.12)	(0.11)	(0.11)	(0.12)
Age	1.02***	1.01***	1.01***	1.01***
	(0.00)	(0.00)	(0.00)	(0.00)
Female sex	0.92***	0.93***	0.93***	0.95**
	(0.02)	(0.02)	(0.02)	(0.02)
Year	1.04***	1.05***	1.05***	1.04***
	(0.01)	(0.01)	(0.01)	(0.01)
Charlson index		1.28***	1.28***	1.28***
		(0.01)	(0.01)	(0.01)
At least two infections at once			1.29*** (0.05)	1.27*** (0.07)
Bloodstream infection				1.06
				(0.04)
Pneumonia				0.84***
				(0.04)
Skin and soft tissue infection				1.03 (0.05)
Urinary tract infection (reference case)				–
Constant	0.00***	0.00***	0.00***	0.00***
	(0.00)	(0.00)	(0.00)	(0.00)
Observations	704,115	704,115	704,115	704,115

Exponentiated coefficients; Standard errors in parentheses
First part: Logistic regression, Second part: Negative Binomial regression. SA=Sensitivity analysis
* $p < 0.05$, ** $p < 0.01$, *** $p < 0.001$

Table A3 Sensitivity analysis: Marginal effects from TPM on total days of LTSL

	SA: TPM Model 5	SA: TPM Model 6	SA: TPM Model 7	SA: TPM Model 8
Antibiotic resistance	7.22***	7.06***	6.58***	9.66***
	(0.58)	(0.61)	(0.62)	(0.64)
Age	0.19***	0.17***	0.17***	0.15***
	(0.01)	(0.01)	(0.01)	(0.01)
Female sex	-0.41*	-0.44*	-0.41*	0.16
	(0.20)	(0.19)	(0.19)	(0.19)
Year	0.06	0.13*	0.12	0.13*
	(0.07)	(0.06)	(0.06)	(0.06)
Charlson index		0.86***	0.83***	0.89***
		(0.08)	(0.08)	(0.08)
At least two infections at once			7.01***	1.33**
			(0.39)	(0.51)
Bloodstream infection				7.64***
				(0.38)
Pneumonia				5.80***
				(0.39)
Skin and soft tissue infection				3.81***
				(0.40)
Urinary tract infection (reference case)				–
Observations	704,115	704,115	704,115	704,115

Standard errors in parentheses. SA=Sensitivity analysis
* $p < 0.05$, ** $p < 0.01$, *** $p < 0.001$

Table A4 Sensitivity analysis: First and second part of TPM with Poisson regression on net days of LTSL

	SA: TPM<br>Model 9	SA: TPM<br>Model 10	SA: TPM<br>Model 11	SA: TPM<br>Model 12
**Part 1: Odds ratio (OR)**				
Antibiotic resistance	1.46***	1.51***	1.43***	2.23***
	(0.06)	(0.06)	(0.06)	(0.10)
Age	1.01***	1.01***	1.01***	1.01***
	(0.00)	(0.00)	(0.00)	(0.00)
Female	1.04**	1.02	1.02	1.09***
	(0.01)	(0.01)	(0.01)	(0.01)
Year	0.97***	0.97***	0.97***	0.97***
	(0.00)	(0.00)	(0.00)	(0.00)
Charlson index		0.86***	0.86***	0.87***
		(0.00)	(0.00)	(0.00)
At least 2 simultaneous infections			2.01***<br>(0.06)	0.93*<br>(0.03)
Bloodstream infection				2.74***
				(0.06)
Pneumonia				2.75***
				(0.06)
Skin and soft tissue infection				1.65***<br>(0.04)
Urinary tract infection (reference case)				–
Constant	2.18e+25***	1.10e+28***	3.82e+28***	2.36e+24***
	(1.82e+26)	(9.09e+28)	(3.17e+29)	(1.96e+25)
**Part 2: Incidence Rate Ratios (IRR)**				
Antibiotic resistance	1.95***	1.73***	1.69***	1.68***
	(0.14)	(0.12)	(0.12)	(0.13)
Age	1.02***	1.01***	1.01***	1.01***
	(0.00)	(0.00)	(0.00)	(0.00)
Female	0.88***	0.88***	0.89***	0.91***
	(0.02)	(0.02)	(0.02)	(0.02)
Year	1.04***	1.05***	1.04***	1.04***
	(0.01)	(0.01)	(0.01)	(0.01)
Charlson index		1.22***	1.22***	1.22***
		(0.01)	(0.01)	(0.01)
At least 2 simultaneous infections			1.30***<br>(0.06)	1.19**<br>(0.08)
Bloodstream infection				1.11*
				(0.05)
Pneumonia				0.83***
				(0.04)
Skin and soft tissue infection				1.13*<br>(0.06)
Urinary tract infection (reference case)				–
Constant	0.00***	0.00***	0.00***	0.00***
	(0.00)	(0.00)	(0.00)	(0.00)
Observations	704,115	704,115	704,115	704,115

Exponentiated coefficients; Standard errors in parentheses
First part: Logistic regression, Second part: Poisson regression. SA=Sensitivity analysis
* $p < 0.05$, ** $p < 0.01$, *** $p < 0.001$

Table A5 Sensitivity analysis: Marginal effects from TPM with Poisson regression on net days of LTSL

	SA: TPM Model 9	SA: TPM Model 10	SA: TPM Model 11	SA: TPM Model 12
Antibiotic resistance	5.93***	5.33***	4.90***	7.18***
	(0.47)	(0.47)	(0.47)	(0.49)
Age	0.15***	0.13***	0.13***	0.11***
	(0.01)	(0.01)	(0.01)	(0.01)
Female	-0.59***	-0.62***	-0.59***	-0.14
	(0.17)	(0.16)	(0.16)	(0.16)
Year	0.06	0.08	0.07	0.06
	(0.06)	(0.05)	(0.05)	(0.06)
Charlson index		0.39***	0.37***	0.40***
		(0.04)	(0.04)	(0.04)
At least 2 simultaneous infections			5.19*** (0.33)	0.63 (0.42)
Bloodstream infection				5.87***
				(0.31)
Pneumonia				4.20***
				(0.33)
Skin and soft tissue infection				3.32*** (0.34)
Urinary tract infection (reference case)				–
Observations	704,115	704,115	704,115	704,115

Standard errors in parentheses
First part: Logistic regression, Second part: Poisson regression. SA=Sensitivity analysis
* $p < 0.05$, ** $p < 0.01$, *** $p < 0.001$
